# ElucidatiNg Immunosuppressant pharmacokinetic variabilities by investigating Gut Microbiome modulations After kidney transplantation (ENIGMA): study protocol of a prospective longitudinal trial

**DOI:** 10.1136/bmjopen-2025-106623

**Published:** 2026-06-07

**Authors:** Alice Barbé, Lidvine Boland, Nada Kanaan, Tom Darius, Antoine Buemi, Yannick France, Marie-Agnès Ronsyn, Sarah Rahal, Maxime Lingurski, Françoise Van Bambeke, Laure B. Bindels, Vincent Haufroid, Arnaud Devresse, Laure Elens

**Affiliations:** 1 Department of Integrated PharmacoMetrics, PharmacoGenetics and PharmacoKinetics Group, Louvain Drug Research Institute, Université catholique de Louvain, Brussels, Belgium; 2 Cellular and Molecular Pharmacology Group, Louvain Drug Research Institute, Université catholique de Louvain, Brussels, Belgium; 3 Metabolism and Nutrition Research Group, Louvain Drug Research Institute, Université catholique de Louvain, Brussels, Belgium; 4 Department of Clinical Chemistry, Cliniques universitaires Saint-Luc, Brussels, Belgium; 5 Louvain Center for Toxicology and Applied Pharmacology, Institut de Recherche Expérimentale et Clinique, Université catholique de Louvain, Brussels, Belgium; 6 Department of Nephrology, Cliniques universitaires Saint-Luc, Brussels, Belgium; 7 Nephrology Department, Institut de Recherche Experimentale et Clinique, Université catholique de Louvain, Brussels, Belgium; 8 Surgery and Abdominal Transplant Unit, Cliniques universitaires Saint-Luc, Brussels, Belgium; 9 WELBIO Department, WEL Research Institute, Wavre, Belgium; 10 Department of Nephrology, Cliniques universitaires Saint-Luc, Bruxelles, Belgium

**Keywords:** CLINICAL PHARMACOLOGY, Renal transplantation, Gastrointestinal Microbiome, Antibiotics, Immunity

## Abstract

**Introduction:**

In kidney transplantation, immunosuppressive therapy is essential to control alloimmune reactions, prevent graft rejection and improve patient survival rates. However, commonly used drugs like tacrolimus (TAC) and mycophenolate mofetil (MMF) have a narrow therapeutic window and exhibit significant inter- and intra-individual variability in pharmacokinetics (PK) and dose-response relationships. Recent pilot studies suggest that the gut microbiome may influence this variability.

**Methods and analysis:**

ElucidatiNg Immunosuppressant pharmacokinetic variabilities by investigating Gut Microbiome modulations After kidney transplantation (ENIGMA) is a prospective, low-interventional, naturalistic longitudinal trial designed to identify biomarkers of TAC and MMF PK variability by examining gut microbiome changes and modulations after kidney transplantation and their link with TAC and MMF PK. Biological samples from 50 patients will be collected at nine specific timepoints pre- and post-transplantation using a rich PK and biological sampling strategy. This approach will enable the derivation of PK parameters for the investigated drugs and the creation of a biobank for future hypothesis testing.

**Ethics and dissemination:**

The ENIGMA trial has received ethical approval from the European Medicines Agency (EMA). The reference number of our project is R&D/1325226 and is registered on the Clinical Trial Information System (CTIS) platform with European Union Clinical Trial number 2023–5 08 335-31-00. Results of the trial will be published in scientific journals and presented at different (inter)national conferences.

**Trial registration number:**

2023–5 08 335-31-00 EMA.

**STRENGTHS AND LIMITATIONS OF THIS STUDY**

- Longitudinal design enables in-depth analysis of the relationship between inter- and intra-patient variability in tacrolimus (TAC) and mycophenolate mofetil (MMF) pharmacokinetics (PK) and microbiome dynamics.
- Integration of at-home volumetric absorptive microsampling in a substudy allows for supplementary TAC and MMF PK data collection, enhancing real-world applicability.
- Collection of both pre- and post-transplantation samples provides a comprehensive view of temporal biological changes.
- Extensive biobanking ensures a rich repository of biological samples for future translational research.
- Limited sample size (n=50) may restrict the statistical power and generalisability of findings.

## Introduction

 Kidney transplantation is the main treatment option for chronic end-stage kidney failure if the patient is suitable for surgery.^1^ Thanks to immunosuppressive therapies of increasing efficacy, the survival of grafts has been constantly progressing over the last decades.^2^ However, the long-term survival rates still need to be improved as maximising long-term graft survival will allow to delay the moment patients will need further transplants, thereby reducing the number of patients on waiting lists.^3^ While the incidence of acute rejection episodes has decreased,^4^ long-term immunosuppression complications have become increasingly evident.^5^

One of the main modifiable risk factors in graft survival is the control of immunosuppression.^3 6 7^ Maintenance therapy currently typically consists of a triple-drug regimen including corticosteroids, a calcineurin inhibitor, tacrolimus (TAC), and an anti-metabolite, mycophenolate mofetil (MMF).^8^ Maintaining optimal levels of immunosuppression while avoiding over- and/or under-dosage due to unexplained pharmacokinetic (PK) variability is essential to prevent increased toxicity and allograft rejection.^9 10^ However, TAC and MMF are known narrow therapeutic-toxic margin drugs and exhibit high interindividual and intraindividual dose-response variations. For TAC, this has led to the implementation of therapeutic drug monitoring (TDM) to avoid inadequate drug levels, thereby minimising the risk of rejection and adverse drug reactions.

Although TDM remains central to the management of TAC-based immunosuppression, it is not routinely used for MMF and constitutes a reactive rather than proactive approach, lacking clear guidelines. For TAC, studies have shown that only 18.5%–37.4% of patients achieve therapeutic levels at first steady state,^11^,^13^ prompting researchers to explore new biomarkers of PK variability. Despite over 1000 publications on TAC dosage requirements depending on the *CYP3A* genotype, mainly CYP3A5*3 and, to a lesser extent CYP3A4*22,^14^ the implementation of pre-emptive genotyping and Clinical Pharmacogenetics Implementation Consortium (CPIC) dosing guidelines^15^ is not widespread in transplant centres, likely due to insufficient evidence of benefits on graft and patient outcomes, a lack of strong guidelines and/or cost.^16^ There is a clear need for new PK biomarkers to improve follow-up care for renal transplant patients.

Previous work has allowed understanding a part of the inter-patient variations in terms of dose-response of TAC and mycophenolic acid (MPA), the active metabolite of MMF. The influence of patient’s genetic profile can explain part of the observed PK variability.^17^ For instance, patients presenting different *CYP3A5* phenotypes show an important shift in TAC metabolism which accounts for approximately 50% of the observed dose-requirement variation and has led to the abovementioned CPIC genotype-based dosing guidelines. For MPA, variants in *UGT1A9* and *ABCC2* have been associated with differences in PK parameters without clear pharmacogenetic testing guidelines or interpretation.^9^ Other factors of inter-patient variations include age at transplantation, co-medications, body surface area and others.^9 17^ More recently, the gut microbiota composition has been shown to be an important factor of inter-individual PK variability.

The importance of the gut microbiome for explaining the fate of immunosuppressive drugs in the organism has been largely understudied although it is now widely accepted that the complex ecosystem of intestinal microbes dynamically takes part in several functions of the host beyond digestion, including drug metabolism.^18^,^22^ Indeed, the gut microbiota synthesises numerous compounds that may interact with and affect the human host metabolome. Not only gut microorganisms express numerous enzymes able to directly metabolise xenobiotics^23^ but they are also able to control the host PK phenotype through different undirect processes.^24^ Inter-individual differences in PK profiles might thus be explained by between-subject differential microbiota composition or function. Moreover, contrarily to the host genetic make-up, the microbiota is not inflexible and might thus be a source of intra-individual variability. Microbiota composition is in fact affected by many environmental factors such as diet, lifestyle, age, physiological status and pharmaceuticals.^25 26^ Studies have highlighted differences in gut microbiota composition of transplanted patients compared with healthy patients,^27^,^29^ with significant changes also observed in the microbiome of patients’ pre-transplantation samples compared with post-transplantation samples,^30^,^32^ indicating a potential interplay between microbiota, transplant status and immunosuppressive pharmacotherapy.

The investigation into the role of the gut microbiome in TAC PK has commenced, with various in vitro and in vivo studies yielding interesting insights. In vitro, Guo *et al* suggest that *F. prausnitzii* metabolises TAC into a less potent immunosuppressive metabolite.^33^ Preclinical results in mice show that microbiota depletion increases *Abcb1a* intestinal expression, leading to a decreased TAC absorption. Additionally, repeated TAC administration also significantly alters gut microbiota composition at various taxonomic levels.^34^ In clinics, gut microbiota composition of TAC-treated renal transplant patients has been associated with different dose requirements in CYP3A5 poor metabolisers.^35^ This effect may be due to the influence of the gut microbiota on the activity of genes important in TAC PK (eg, *ABCB1*). Conversely, gut microbiota might be affected differently by escalating TAC dose.^36^

The gut microbiome is implicated in MPA metabolism, as MPA undergoes enterohepatic recirculation where its inactive metabolite, MPA 7-O-glucuronide, is hydrolysed by bacterial β-glucuronidases to regenerate MPA. MMF has been reported to alter the composition of the gut microbiota by selecting for bacteria expressing β-glucuronidase leading to an upregulation of β-glucuronidase activity in the gut of mice and patients presenting gastrointestinal (GI) side effects.^37^ Additionally, it has been shown that vancomycin eliminated β-glucuronidase expressing bacteria and prevented MMF GI toxicity in mice.^37^ In the same line, another study showed that an intact gut microbiota is required to initiate and sustain the MMF GI toxicity.^38^ From a PK point of view, studies in healthy volunteers treated with cholestyramine have demonstrated that the interruption of the enterohepatic recirculation decreases MPA exposure by approximately 40%.^39^ Furthermore, it has been shown that the inhibition of ABCC2-mediated hepatic excretion paradoxically decreases MPA exposure.^40^ ABC transporter expression modulation by the gut microbiota might be another source of MPA(G) PK variability.

Given the evident interaction between immunosuppressive drugs and the gut microbiome, other medications that affect the gut microbiota may indirectly influence the PK of these drugs. Antibiotics, in particular, have a significant impact on the gut microbiota.^41^ Additionally, transplant recipients are likely to use these drugs due to infections resulting from their compromised immune system.

Immunosuppressant PK variability continues to challenge optimal treatment response in kidney transplant patients. Graft survival and drug-related toxicities remain common clinical issues. Understanding the factors behind treatment response variability is crucial for individualised treatment strategy. Building on previous studies that have shown a connection between the gut microbiome and the response to immunosuppressive therapy in kidney transplant patients, the next step is to explore and characterise how the gut microbiome affects the PK of the two most commonly used immunosuppressive drugs in these patients, with the aim of identifying new biomarkers.

## Hypothesis and objectives

The hypothesis of the trial is that the gut microbiota composition, by inducing physiological changes, is influencing both the oral absorption of TAC and the entero-hepatic recirculation of MPA, thereby impacting on their PK profile, and possibly explaining the variability in the treatment response.

The primary aim of the ENIGMA trial is to identify staging biomarkers of TAC and MMF PK variability by investigating the gut microbiota modulations and physiological changes following kidney transplantation. Additionally, the trial seeks to link PK variabilities, microbiome changes overtime (metabolic and compositional) and responses to immunosuppressive therapy (such as graft survival/rejection and drug-related toxicities). Given the common use of antibiotics in this population, the study will also assess the effect of antibiotherapy interventions on the microbiota composition and the subsequent consequences on immunosuppression pharmacotherapy.

As a secondary endpoint, the study will also explore whether the genotype of the donor and the recipient can predict PK variabilities. Furthermore, it will analyse the response to therapy and related physiological changes from a molecular perspective (ie, transcriptomics, metabolomics and proteomics). Ultimately, this trial aims to link gut microbiota changes and PK variabilities to other blood and urinary-omics data to evaluate their potential as biomarkers and elucidate the underlying mechanisms of the arising observations.

## Method and analysis

### Study design

ElucidatiNg Immunosuppressant pharmacokinetic variabilities by investigating Gut Microbiome modulations After kidney transplantation (ENIGMA) is a single-centre, prospective, low-interventional, naturalistic, longitudinal trial in which 50 living donor kidney transplant recipients are followed for 14 months (European Union Clinical Trial (EUCT) number 2023–5 08 335-31-00). The patients receive standard of care immunosuppressive therapy consisting of TAC (Advagraf), MMF (Cellcept) and corticosteroids. Each patient is enrolled approximately 2 months before kidney transplantation and followed for 12 months post-surgery.

All living kidney donors are managed according to the standard procedures required in the accredited transplant centre and in line with the international guidelines.^42^ Donors are not participants in this clinical trial, and their care is not modified by the study. Before donation, donors undergo a comprehensive medical assessment, as well as informed consent procedures. During the donation procedure, perioperative management follows established institutional protocol for living donor nephrectomy. After donation, donors receive routine postoperative follow-up as recommended by international guidelines, including early postoperative care, monitoring of renal function and long-term follow-up visits.

**Figure 1:**
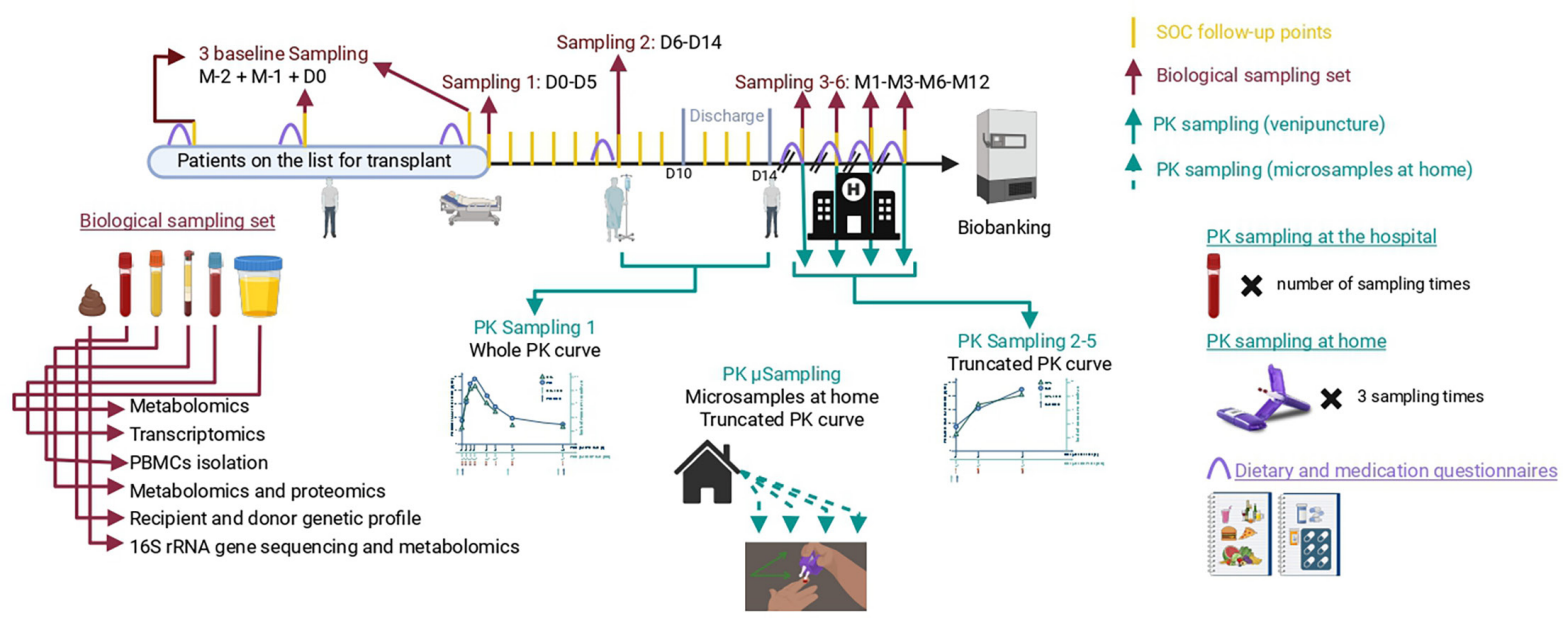
ElucidatiNg Immunosuppressant pharmacokinetic variabilities by investigating Gut Microbiome modulations After kidney transplantation (ENIGMA) longitudinal trial design. D, day; M, month; PBMC, peripheral blood mononuclear cells; PK, pharmacokinetic; SOC, standard of care; µsampling, microsampling.

The trial is designed in three different periods: pre-transplant, hospitalisation and ambulatory period (figure 1). During these visits, a complete biological sampling set including blood, urine and faeces is collected. The different blood collection tubes are Li Heparin LH/7.5 mL (Sarstedt) for proteomic and metabolomic analysis, EDTA K3E/3.4 mL (Sarstedt) for pharmacogenetic testing and immunosuppressants blood concentration measurements, Tempus Blood RNA tubes (Thermo Fisher Scientific) for transcriptomic analysis, BD Vacutainer CPT Mononuclear Cell Preparation Tube – Sodium Heparin (BD Biosciences) for peripheral mononuclear blood cell (PBMC) isolation and Cell-Free DNA BCT (Streck) for donor-derived cell-free DNA analysis. The faecal sample is accompanied by a dietary and medication questionnaire. During the follow-up, a food frequency questionnaire will be carried out twice (once for period before transplantation, once for period post-transplantation).

Concerning the preanalytical treatment of the collected samples, plasma is separated in heparinized tubes after centrifugation (2600 g for 10 min at 4°C). PBMCs are isolated according to manufacturer’s protocol. Cell-free DNA is isolated according to manufacturer’s protocol as well. All samples are aliquoted and snap freezed in liquid nitrogen before storage at −80°C within 2 hours after collection. Faeces are frozen at −20°C immediately after sampling and transferred to −80°C within 3 days after collection. Whole blood for TAC quantification is stored at 4°C before analysis. MPA blood samples are collected on ice in a cold transport container and centrifuged at 871 g for 10 min at 4°C. Plasma is then acidified by addition of orthophosphoric acid 85% (1/50) and stored at −80°C before MPA and metabolite quantification.

Depending on the visit, the composition of the set varies slightly. Stool, urine and blood samples are collected at every visit. The EDTA whole blood sample used for pharmacogenetic testing is collected once at the M-2 visit. PBMCs are collected starting from the D0 visit to study the intracellular concentration of immunosuppressive drugs. A Tempus Blood RNA tube is collected at every other visit post-transplantation. The Cell-free DNA BCT tube is collected once at the M12 visit.

**Figure 2:**
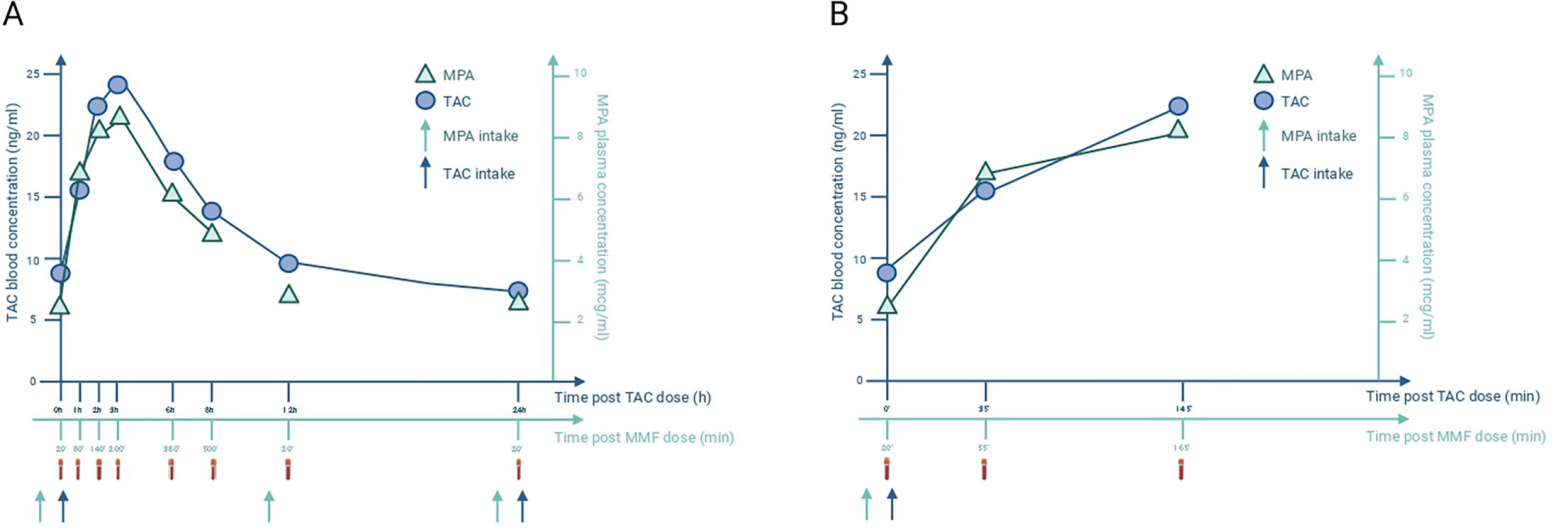
(A) Full pharmacokinetic curve scheme. (B) Truncated pharmacokinetic curve scheme. h, hours; min, minutes; MPA, mycophenolic acid; TAC, tacrolimus.

In addition, two types of PK profiling are performed to assess the drug exposure through two different approaches: a full PK curve time course during hospitalisation and a truncated PK curve at different occasions in an ambulatory setting. The full PK curve consists of eight sampling time points in 24 hours (at 0, 1, 2, 3, 6, 8, 12 and 24 hours after TAC intake). MPA is taken 20 min before the first time point (figure 2A). The truncated PK curve consists of three sampling time points (at 0, 35 min and 2 hours and 25 min after TAC intake) (figure 2B). MPA is again taken 20 min before the first time point.

#### Pre-transplant period

Standard pre-transplant visits allow to plan three baseline biological material collection points, 2 months, 1 month and just before transplantation when the patient is hospitalised. During these three visits, a biological sampling set is collected.

#### Hospitalisation period

During the hospitalisation period, samples are collected directly after transplantation (between days 0 and 5) and as from day six after transplantation. The full PK curve is sampled as from day six as well.

#### Ambulatory period

In the standard of care, after discharge of the hospital, the patient visits the hospital regularly. Biological sampling collection takes place at four of these visits, at 1, 3, 6 and 12 months post-transplantation. A truncated PK curve is sampled at each of these four visits.

### Antibiotics

The study also intends to explore the PK of antibiotics usually administered in the management of infections in kidney transplant patients. Its relationship with microbiota modulations and the consequences on immunosuppressive drug PK and clinical outcomes will be tested as well. Therefore, if, for any medical reason (eg, infection), the patient must initiate an antibiotic therapy, and an additional set of biological samples after this medication change is collected. This visit consists of collecting various biological samples (as mentioned in *Study design*) and performing a truncated PK curve.

### Volumetric absorptive microsampling substudy

With the aim to collect complementary PK data, patients are invited to take part in a substudy that involves the drawing of blood samples at home using a volumetric absorptive microsampling device. The same scheme of sampling as for ambulatory visits is scheduled at home (C_0h_-C_35min_-C_2h25_ for TAC corresponding to C_20min_-C_55min_-C_165min_ for MPA). The patient is informed about the possibility to benefit from this follow-up at home during the study inclusion. If he/she agrees to provide microsampling measurements, he/she is trained to use the Mitra Microsampling Device during the hospitalisation period under the supervision of trial staff. Per patient, maximum four truncated PK curves are sampled at home in the 1-year post-transplantation period.

### Study population

The study population will include 50 kidney transplant patients planned for a living-donor kidney transplant. All patients are aged >18 years old and <75 years old with a body mass index ranging from 18 to 35 kg/m^2^. Both ABO-compatible and incompatible situations are considered for inclusion. As inflammatory bowel diseases (IBD) and bariatric surgery are known to be linked to altered microbiota composition and function; no patients suffering from IBD or having undergone bariatric surgery are considered for participation to the study because of the risk of a strong confounding effect. The full list of inclusion and exclusion criteria is available in box 1.

Box 1 **Full inclusion and exclusion criteria of the ElucidatiNg Immunosuppressant pharmacokinetic variabilities by investigating Gut Microbiome modulations After kidney transplantation (ENIGMA) trial**

**Inclusion criteria**

Diagnosis of chronic renal failure

Planned for a kidney transplantation

Receiving a living-donor kidney

Followed at Cliniques Universitaires Saint-Luc

Immunosuppressive treatment consisting in a combination of Tacrolimus (Advagraft) and mycophenolate mofetil (Cellcept)

Aged between 18 and 75 years old at time of enrolment

BMI ranging from 18 to 35 kg/m^2^

Not being pregnant

**Exclusion criteria**

Diagnosis of IBD

History of bariatric surgery

Alcohol or drug addiction

Aged<18 or >75 years old

BMI lower than 18 or higher than 35 kg/m^2^ kg/m2

Pregnancy

Not able to provide informed consent

BMI, body mass index, IBD, inflammatory bowel disease

#### Primary and secondary outcomes

The primary endpoints are related to immunosuppressive drug (including TAC, MPA and metabolites) and antibiotic PK parameters. These include but will not be limited to drug and metabolite blood exposure as assessed by the trough concentration (C_0h_) or area under the curve (AUC_0-t_) (extrapolated or calculated), drug dosage, PBMC drug concentration and accumulation and drug/metabolite concentrations in faecal material.

Microbiota composition and function are also considered as primary outcomes. This includes diversity estimation and bacterial relative abundance determination as assessed by 16S ribosomal ribonucleic acid (rRNA) gene amplicon sequencing, virome characterisation as assessed by shotgun metagenomics and/or a targeted approach and faecal metabolomic profiling.

Secondary outcomes consist of markers of drug-related toxicity, markers of graft rejection, markers of antibiotherapy efficacy and patient-omic profiling (see table 1 for non-exhaustive specified secondary outcomes).

**Table 1:** Secondary outcomes of the ElucidatiNg Immunosuppressant pharmacokinetic variabilities by investigating Gut Microbiome modulations After kidney transplantation (ENIGMA) trial

Secondary outcomes			
Markers of drug-related toxicity	Markers of graft function and rejection	Markers of antibiotherapy efficacy	Patient -omic profiling
Diabetes mellitus	Creatinine	Resistance	Blood transcriptomics
Hypertension	Glomerular filtration rate	Recurrence	Plasma and urinary metabolomics
Renal toxicity	Graft functional biomarkers		Blood and urinary lipidomics
Diarrhoea and GI toxicity	Per protocol biopsy and histologic anatomopathological evaluation		Plasma proteomics
Neurotoxicity	Circulating donor cell-free DNA measurement		Whole exome sequencing
Infections	Donor-specific antibodies (DSA)		
Tumours			

GI, gastrointestinal.

### Sample size calculation

Power calculation indicates that a sample size of 50 patients with nine repeated PK measurements will provide an 80% statistical power considering a type I (alpha error) of 5% to detect clinically relevant results. The power to detect true associations strongly depends on effect size, heterogeneity of the background noise and sample size. The noise signal is limited by (i) anticipating baseline measurements, (ii) selecting living-donor recipient (limits the variability in phenotypes), (iii) monitoring the individuals over a long period of time (ie, 12 months) and (iv) detaching the interoccasion from the intraoccasion variability.

Considering an SD of 57.5 ng/mL per mg/kg in the TAC dose requirement (preliminary data) and a repeated measurement design with 9 PK time points, a power of 0.8 (1-β = π=80%) can be guaranteed to detect a Δmean as low as 17.25 ng/mL per mg/kg across the levels of a tested-independent variable with a typical type I (α) error of 0.05 (ie, effect size=0.30) which correspond to an average ΔCmin of 2.6 ng/mL considering a typical standard dose (ie, 0.15 mg/kg). This difference can be considered as clinically relevant (therapeutic target 7–20 ng/mL).

Considering a SD of 2.6 µg/mL in the MPA concentrations for a typical dose of 1000 mg (preliminary data) and a repeated measurement design with 9 PK time points, a power of 0.8 (1-β = π=80%) can be guaranteed to detect a Δmean as low as 0.78 µg/mL across the levels of a tested independent variable with a typical type I (α) error of 0.05 (ie, effect size=0.30). Considering that the range of MPA exposure varies between ±2 to 10 µg/mL depending on the time post-dose, this difference can also be considered as clinically relevant.

### Data management

Patients’ medical data—including demographics, medical history, treatments and laboratory results—are collected from their medical records at the time of inclusion and continuously throughout the entire follow-up period. Both standard-of-care and study-specific data are securely stored using Research Electronic Data Capture, a web-based software platform designed to support data capture for research studies. Access to the platform is restricted to authorised personnel to ensure confidentiality and data integrity. The collected data will be archived for 25 years.

### Data analysis

The different PK phenotypes will be analysed either as continuous variable or categorical variables (classified according to the percentile range). These PK phenotypes include primarily classical PK parameters (clearance, absorption rate, AUC, drug systemic concentrations at a given time point), intra-patient variability index as assessed by the coefficient of variation observed in the dose-adjusted concentrations across the follow-up, for MPA, the metabolite to parent drug ratio, dose requirement (ie, dose adjusted drug exposure), number of dose adjustments needed to achieve stable target levels and time to achieve stable target levels.

### Study discontinuation

Patients will be withdrawn from the trial’s follow-up if they enter one or more of the conditions mentioned in box 2.

Box 2 **Conditions for patient withdrawal from ElucidatiNg Immunosuppressant pharmacokinetic variabilities by investigating Gut Microbiome modulations After kidney transplantation (ENIGMA) trial**

**Conditions for patient withdrawal from ENIGMA**

Pregnancy

Immunosuppressive therapy switch during follow-up (for any reasons)

At the end of follow-up

Voluntary discontinuation

Suspected noncompliance

Death

Graft loss

Data collected before patient withdrawal or discontinuation will be kept and included in the data analysis unless not authorised by the patient or suspected to confound the results. No additional data will be generated after patient withdrawal and his/her follow-up will be stopped.

## Ethics, trial status and dissemination

This trial is being conducted in accordance with the protocol (third version, 27 October 2025), Good Clinical Practice, the Declaration of Helsinki and the moral, ethical and scientific principles that justify medical research. Organs will be sourced ethically. Organs will not be sourced from executed prisoners or prisoners of conscience or other vulnerable groups. The ENIGMA trial has received ethical approval from the European Medicines Agency and is registered on the Clinical Trial Information System platform with EUCT number 2023–5 08 335-31-00.

The recruitment of patients started in September 2024.

Results of the trial will be published in scientific journals and presented at different (inter)national conferences. Individual participant data will not be shared. Participants have given their approval for the pseudomised study data sharing according to the consent form version on the informed consent form approved version log of the study. In case of request to share study data by external parties, there is a secure transfer and anonymised system, and a data processing agreement must be signed between both parties. In accordance with findability, accessibility, interoperability, and reusability (FAIR) data principles, all raw and processed data will be shared in standard community formats, thoroughly annotated and deposited in appropriate repositories (eg, ProteomeXchange, MetaboLights and European Nucleotide Archive). Software and analysis pipelines will be fully documented and released under permissive open-source licences on publication.

## Discussion

Better understanding of immunosuppressant drug PK will permit to avoid or limit drug under-exposure and over-exposure which are linked to graft rejection and drug toxicity, respectively. Theoretically, such a strategy will lead to reduced number of graft rejections and re-transplantations on the one hand and on side effects the other hand. As such, utilisation of prescribing algorithms that incorporate biomarkers predictive of a specific outcome in clinical practice has the potential to reduce the TDM, hospitalisation and pharmacological costs to increase patient adherence and to improve the patient’s quality of life.

From a societal point of view, patient recovery would be positively impacted by promoting a steady and fast reintegration into his/her active life. It will further help the clinicians in decision-making and provide a more comprehensive understanding of the clinical problem, thereby easing healthcare management and alleviating medical burden.

More broadly, our study will provide the scientific community with important data to better understand the interaction between immunosuppressant pharmacology and gut microbiome from a clinical perspective, potentially advancing precision pharmacotherapy. Given that various biotransformation enzymes and transporters have a wide overlapping range of substrates, confirming a link between microbiota composition and PK functions could allow findings to be extrapolated to other drugs and diseases.

### Patient and public involvement

The informed consent form template used has been approved by representatives of patient organisations.
