# Supplementary material for: ElucidatiNg Immunosuppressant pharmacokinetic variabilities by investigating Gut Microbiome modulations After kidney transplantation (ENIGMA): study protocol of a prospective longitudinal trial
Source: BMJ Open. 2026 Jun 7;16(6):e106623. doi: 10.1136/bmjopen-2025-106623 (PMC13250183; doi:10.1136/bmjopen-2025-106623)
Supplement: Supplementary file 1 [file bmjopen-2025-106623.reviewer_comments.pdf]

PEER REVIEW HISTORY

BMJ Open publishes all reviews undertaken for accepted manuscripts. Reviewers are asked to complete a checklist review form and are provided with free text boxes to elaborate on their assessment. These free text comments are reproduced below.

ARTICLE DETAILS

Title (Provisional)

ElucidatiNg Immunosuppressant pharmacokinetic variabilities by investigating Gut Microbiome modulations After kidney transplantation (ENIGMA): study protocol of a prospective longitudinal trial

Authors

Barbé, Alice; Boland, Lidvine; Kanaan, Nada; Darius, Tom; Buemi, Antoine; France, Yannick; Ronsyn, Marie-Agnès; Rahal, Sarah; Lingurski, Maxime; Van Bambeke, Françoise; Bindels, Laure; Haufroid, Vincent; Devresse, Arnaud; Elens, Laure

---

VERSION 1 - REVIEW

**Reviewer**	**1**
**Name**	**M Gonzalez, Fernando**
**Affiliation**	**University of Chile, Nephrology**
**Date**	**17-Nov-2025**
**COI**	**None**

The project is very well formulated. The manuscript has been carefully redacted and is clear.

Nevertheless, I think that the expected crude results, that microbiome can modify tacrolimus and mycophenolic acid blood levels, will be surely demonstrated, but it is not clear if those results will change physicians' behaviour about immunosuppressants drug prescription.

Moreover, as the tacrolimus formulation chosen as immunosuppressive is a once a daily one (Advagraf), it is less probable that physicians take account of the probable pharmacokinetic variability of blood drug levels, Even when coprescribing antibióticos.

I suggest adding some measurement related to valoration of the theoretical pharmacokinetic changes by the clinical transplant team.

VERSION 1 - AUTHOR RESPONSE

Reviewers’ Comments to the Author

- The project is very well formulated. The manuscript has been carefully redacted and is clear. Nevertheless, I think that the expected crude results, that microbiome can modify tacrolimus and mycophenolic acid blood levels, will be surely demonstrated, but it is not clear if those results will change physicians' behaviour about immunosuppressants drug prescription.
    - We thank the reviewer for this thoughtful comment. The interaction between gut microbiome modulations and immunosuppressant drugs has indeed been investigated in multiple scientific studies, including those summarized in the review by Mohamed et al. (Transplantation, 2024). The primary objective of our trial is to identify biomarkers associated with tacrolimus and mycophenolate mofetil pharmacokinetic variability by investigating gut microbiota modulations. We agree that the results of this study alone will not immediately modify physicians' prescribing behaviors. However, if robust biomarkers can be identified, they could ultimately contribute to more personalized immunosuppressant dosing strategies in clinical practice.
      The clinical translation of gut microbiota-based approaches is currently being explored across multiple therapeutic areas. For instance, Gilbert et al. (Nature Medicine, 2025) discuss the emerging clinical perspectives of integrating microbiome data into treatment decision-making. A concrete example is irinotecan dosing in colorectal cancer where strategies incorporating gut microbial enzyme activity are being investigated (Kristen et al., EJSO, 2024). As kidney transplantation remains the goal standard treatment for end-stage kidney disease, we hypothesize that incorporating gut microbiome information may provide valuable insights into the pharmacokinetic variability observed with immunosuppressants to avoid adverse events and graft failure.

- Moreover, as the tacrolimus formulation chosen as immunosuppressive is a once a daily one (Advagraf), it is less probable that physicians take account of the probable pharmacokinetic variability of blood drug levels, Even when coprescribing antibiótics.
    - We thank the reviewer for this relevant observation. According to current tacrolimus clinical guidelines, therapeutic drug monitoring is recommended based on Ctrough concentrations, including for once-daily formulations such as Advagraf (Brunet et al., Therapeutic Drug Monitoring, 2019). Consequently, both intra- and inter-individual pharmacokinetic variability are already routinely considered in clinical practice, as tacrolimus dosing is systematically adjusted based on measured Ctrough, regardless of the formulation used. We also note that the current strategy (therapeutic drug monitoring based on Ctrough) is discussed in the manuscript (page 3).

- I suggest adding some measurement related to valoration of the theoretical pharmacokinetic changes by the clinical transplant team.
    - We thank the reviewer for this valuable suggestion. In routine clinical practice, therapeutic drug monitoring for tacrolimus relies on a single Ctrough measurement, while mycophenolate mofetil monitoring is performed only upon specific request. In contrast, our study protocol substantially increases the pharmacokinetic data collected: each participant will provide 20 samples

throughout follow-up instead of the 5 samples typically obtained in standard care. As mentioned above, clinicians currently adjust tacrolimus dosing solely based on Ctrough values. Within this clinical trial, however, the transplant team will not have access to the research PK results, as these measurements are generated exclusively for scientific purposes and not intended to guide clinical decision-making. Nevertheless, we agree that a more comprehensive assessment of drug exposure, such as AUC-based therapeutic drug monitoring could, in the future, provide clinicians with a more accurate understanding of overall immunosuppressant exposure. For example, a patient presenting a low Ctrough but a normal AUC would not require a dose increase, whereas dose adjustment based solely on Ctrough might lead to overtreatment. The extensive PK dataset generated in this study may therefore contribute to future developments in exposure-guided dosing strategies.

We thank the reviewer for these thoughtful and pertinent observations. After careful consideration, we believe that, while these points are important for interpreting and translating the findings into clinical practice, they do not necessitate changes to the prespecified study protocol. The protocol must remain methodologically focused; issues relating to clinical implementation, valuation of pharmacokinetic changes by the transplant team, and potential impacts on prescribing behaviour are more appropriately addressed once empirical results are available. We will carefully consider the reviewer's suggestions when preparing the manuscript reporting the study outcomes, and will explicitly discuss clinical relevance, potential strategies to valorise our findings, and future research directions aimed at informing prescribing practice.

VERSION 2 - REVIEW

**Reviewer**	**1**
**Name**	**M Gonzalez, Fernando**
**Affiliation**	**University of Chile, Nephrology**
**Date**	**11-Feb-2026**
**COI**	

All my suggestions were well addressed by the authors.

I think this final version of the manuscript is OK.
